# The Divergence of Neandertal and Modern Human Y Chromosomes

**DOI:** 10.1016/j.ajhg.2016.02.023

**Published:** 2016-04-07

**Authors:** Fernando L. Mendez, G. David Poznik, Sergi Castellano, Carlos D. Bustamante

**Affiliations:** 1 Department of Genetics, Stanford University, Stanford, CA 94305, USA; 2 Program in Biomedical Informatics, Stanford University, Stanford, CA 94305, USA; 3 Department of Evolutionary Genetics, Max Planck Institute for Evolutionary Anthropology, Leipzig 04103, Germany; 4 Department of Biomedical Data Science, Stanford University, Stanford, CA 94305, USA

## Abstract

Sequencing the genomes of extinct hominids has reshaped our understanding of modern human origins. Here, we analyze ∼120 kb of exome-captured Y-chromosome DNA from a Neandertal individual from El Sidrón, Spain. We investigate its divergence from orthologous chimpanzee and modern human sequences and find strong support for a model that places the Neandertal lineage as an outgroup to modern human Y chromosomes—including A00, the highly divergent basal haplogroup. We estimate that the time to the most recent common ancestor (TMRCA) of Neandertal and modern human Y chromosomes is ∼588 thousand years ago (kya) (95% confidence interval [CI]: 447–806 kya). This is ∼2.1 (95% CI: 1.7–2.9) times longer than the TMRCA of A00 and other extant modern human Y-chromosome lineages. This estimate suggests that the Y-chromosome divergence mirrors the population divergence of Neandertals and modern human ancestors, and it refutes alternative scenarios of a relatively recent or super-archaic origin of Neandertal Y chromosomes. The fact that the Neandertal Y we describe has never been observed in modern humans suggests that the lineage is most likely extinct. We identify protein-coding differences between Neandertal and modern human Y chromosomes, including potentially damaging changes to *PCDH11Y*, *TMSB4Y*, *USP9Y,* and *KDM5D*. Three of these changes are missense mutations in genes that produce male-specific minor histocompatibility (H-Y) antigens. Antigens derived from *KDM5D*, for example, are thought to elicit a maternal immune response during gestation. It is possible that incompatibilities at one or more of these genes played a role in the reproductive isolation of the two groups.

## Introduction

A central goal of human population genetics and paleoanthropology is to elucidate the relationships among ancient populations. Before the emergence of anatomically modern humans in the Middle Pleistocene ∼200 thousand years ago (kya),^1^ archaic humans lived across Africa, Europe, and Asia in highly differentiated populations. Modern human populations that expanded out of Africa in the Upper Pleistocene received a modest genetic contribution from at least two archaic hominin groups, the Neandertals and Denisovans.^2^, ^3^, ^4^, ^5^ Especially in light of hypothesized genetic incompatibilities between Neandertals and modern humans,^6^ it is important to characterize differentiation between their ancestral populations and to investigate potential barriers to gene flow.

When populations diverge from one another, each retains a subset of the variation that existed in the ancestral population. Consequently, sequence divergence times usually exceed population divergence times, and this effect is more pronounced when the ancestral effective population size was large. In humans, a large fraction of genetic diversity is due to ancient polymorphisms that arose long before the emergence of anatomically modern traits. As a result, Neandertal and modern haplotypes are often no more diverged than modern human sequences are among themselves.^2^ This fact complicates the search for introgressed genomic segments, but two features facilitate their detection.^6^, ^7^ First, due to low levels of polymorphism among Neandertals,^5^ introgressed sequences are often quite similar to those of the Neandertal reference. Second, these regions have elevated linkage disequilibrium due to the relatively recent date of admixture, ∼50 kya.^8^, ^9^, ^10^ Although introgressed Neandertal sequences have been identified in modern human autosomes and X chromosomes, no mitochondrial genome (mtDNA) sequences of Neandertal origin have been reported in modern humans, and Neandertal Y-chromosome sequences have not yet been characterized.

Because uniparentally inherited loci have much smaller effective population sizes than autosomal or X-linked loci, the expected differences between sequence and population divergence times are smaller. Therefore, studying these loci can help to delineate an upper bound for the time at which populations last exchanged genetic material. To date, five Neandertal individuals have been whole-genome sequenced to 0.1× coverage or higher,^2^, ^5^ but all were female. Full mtDNA sequences are also available for eight individuals from Spain, Germany, Croatia, and Russia,^11^, ^12^ but the relationship between Neandertal and modern human Y chromosomes remains unknown.

In this work, we analyzed ∼120 kb of exome-captured Y-chromosome sequence from an ∼49,000-year-old (uncalibrated ^14^C)^13^ Neandertal male from El Sidrón, Spain.^14^ We compare it to the human and chimpanzee reference sequences and to the sequences of two Mbo individuals^15^ who carry the A00 haplogroup, the most deeply branching group known.^16^ We identify the relationship between the Neandertal and modern human Y chromosomes and estimate the time to their most recent common ancestor (TMRCA). We also examine coding differences and explore their potential significance for reproductive isolation.

## Material and Methods

### Sequence Data and Processing

We used the Y-chromosome sequences from the exome capture of a Neandertal from El Sidrón, Spain,^14^ and we downloaded the complete sequences of two A00 Y chromosomes.^15^ The Neandertal data included coding, non-coding, and off-target sequences, and all three sequences were mapped against the GRCh37 reference.^14^ Given that the A00 sequences were closely related,^15^, ^16^ we merged them to increase coverage. We called bases for both the Neandertal and A00 sequences by using SAMtools mpileup (v.1.1),^17^ specifying input options to count anomalous read pairs (-A), recalculate base qualities (-E), and filter out poor-quality bases (-Q 17) and poorly mapping reads (-q 20).

We then identified overlapping regions and excluded coordinates with unusually high coverage, filtering out sites with coverage greater than the mean plus five times its square root (Figure S1). Under a Poisson model, this cutoff would elicit the loss of less than one genuine site per 10,000. Finally, we removed sites with inconsistent base calls, discarding those with more than two reads differing from the consensus allele and those for which more than one third of the observed bases did not match the consensus. This filter should minimize the effects of postmortem DNA damage and of modern contamination.

Using the blastz file chrY.hg19.panTro4.net.axt.gz,^18^ we identified the subset of regions within which the human sequences align to the chimpanzee reference. This yielded a total of 118,643 base pairs (bp). In what follows, we refer to this set of sites as “filter 1.” We also identified a second, more restrictive, set of regions totaling 100,324 bp, “filter 2,” by further requiring that the alignment correspond to the chimpanzee Y chromosome rather than to another chimpanzee chromosome (Tables S1A and S1B).

**Figure 1:**
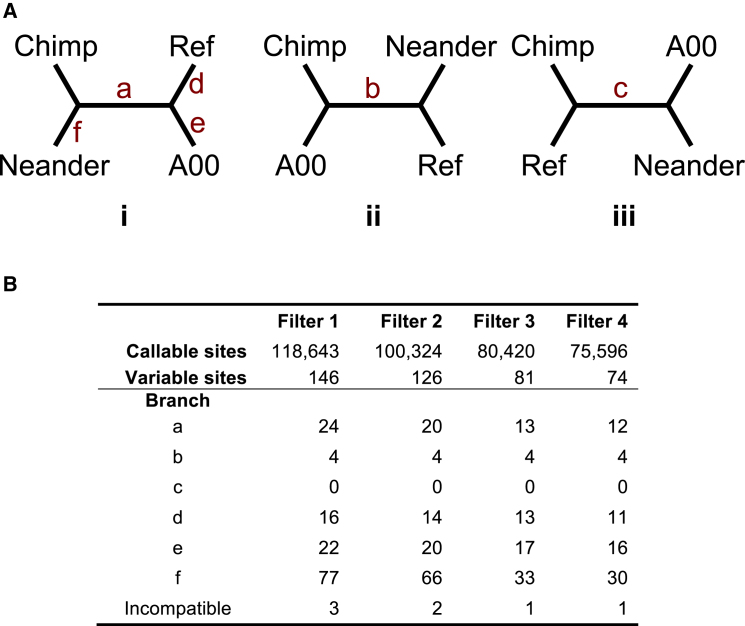
Tree Inference (A) A priori, three trees could feasibly have related the Y chromosomes of the chimpanzee (Chimp), the Neandertal (Neander), haplogroup A00, and the human reference (Ref). Mutations on branch a support topology i, with the Neandertal lineage as the outgroup to those of modern humans, whereas mutations on branches b and c support topologies ii and iii, respectively. Branches d, e, and f correspond to mutations private to individual lineages. (B) Counts of SNVs consistent with each branch. Columns refer to sets of coordinates considered (see Materials and Methods). Incompatible sites are those that cannot be explained by a single mutation on any of the three trees.

For each position within these regions, we determined whether the Neandertal, A00, or both differed from the human reference sequence. We then used the corresponding chimpanzee allele as a proxy for the ancestral state in order to assign the mutation to the appropriate branch of the tree relating the four sequences (Figure 1A). In doing so, we discarded five sites: two at which the chimpanzee carries a third allele, one for which the chimpanzee carries a deletion, and two that were specific to A00 but only supported by a single read. Excluding these sites had little impact on our analyses.

### Estimating TMRCA

**Figure 2:**
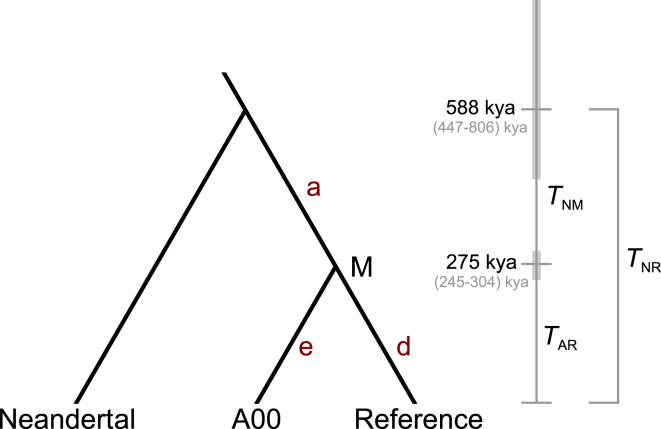
Estimating the TMRCA of Neandertal and Modern Y Chromosomes The quantity of primary interest is *T*_NR_ = *T*_NM_*+ T*_AR_. Branches are labeled as in Figure 1, and “M” denotes the most recent common ancestor of modern human lineages.

To estimate the TMRCA of the Neandertal and modern human Y chromsomes (*T*_NR_), we decomposed this quantity (Figure 2) into the sum of the TMRCA of modern humans (*T*_AR_) and the time separating the most recent common ancestor of modern humans from its common ancestor with the Neandertal lineage (*T*_NM_):

$$T_{\text{NR}}=T_{\text{AR}}+T_{\text{NM}}=\alpha T_{\text{AR}}\alpha \equiv \left(1+\frac{T_{\text{NM}}}{T_{\text{AR}}}\right).$$

We then estimated *T*_AR_ and used two methods to estimate *α.*

To estimate *T*_AR_, we used sequence data from the ancient Ust’-Ishim sample,^9^ first applying the filters described for the A00 sequences. To reduce the potential impact of postmortem DNA damage, we restricted this analysis to coordinates covered by at least three sequencing reads. We further restricted to the subset of Poznik et al.^19^ regions in which the human reference sequence is based on bacterial artificial chromosome clones derived from the RP-11 individual,^20^ a known carrier of haplogroup R1b. This left ∼7.83 Mb of sequence within which to assign variants to the appropriate branches (Figure S2, Appendix A). Using the known age of the Ust’-Ishim individual and the constrained optimization procedure described in Rasmussen et al.,^21^ we obtained parametric bootstrap estimates for *T*_AR_ as well as for the mutation rate and the TMRCA of haplogroup K-M526 (Appendix A). Briefly, we sampled from the process that generated the observed tree (Figure S2) by simulating the number of single nucleotide variants (SNVs) on each branch as a Poisson draw with mean equal to the observed number of mutations. To obtain bootstrap samples of the three parameters, we maximized their joint likelihood for each tree replicate.

In our first approach to estimate *α*, we used the relative numbers of mutations assigned to branches a, d, and e (Figure 1), assigning the four sites that did not fit the consensus topology to the A00 or reference lineages, as appropriate (Appendix A). The proportion of time represented by branch a is:

$$\frac{T_{\text{a}}}{T_{\text{a}}+T_{\text{d}}+T_{\text{e}}}=\frac{T_{\text{NM}}}{T_{\text{NM}}+2T_{\text{AR}}}=\frac{\left(\alpha -1\right)T_{\text{AR}}}{\left(\alpha -1\right)T_{\text{AR}}+2T_{\text{AR}}}=\frac{\alpha -1}{\alpha +1}.$$

Therefore, assuming a time-homogeneous mutation rate, the number of branch-a mutations is binomially distributed with parameters *p* = (*α* – 1) ∕ (*α* + 1) and *n* equal to the total number of mutations. Estimating *p* from the data leads directly to a point estimate and confidence interval (CI) for *α*. This first method has the appealing property that it is independent of both the mutation rate and the absolute values of the times. However, the estimation error might be suboptimal due to uncertainty in both the numerator and the denominator.

In the second method, we estimated *α* via the ratio $T_{\text{NM}}/T_{\text{AR}}$, making use of the fact that we can estimate *T*_AR_ with greater certainty than we can *T*_NM_. To estimate *T*_NM_, we restricted our attention to sequences overlapping the ∼8.8 Mb of sequence analyzed by Karmin et al.,^15^ leaving 80,420 bp (“filter 3”) or 75,596 bp (“filter 4”) when confining our analysis to those sites that passed filter 2 (Figure 1, Tables S2a and S2b). Let *l* equal the total length of sequence under consideration (e.g., 80.42 kb), let *μ* equal the mutation rate over the full 8.8 Mb, let *r* equal the ratio of the mutation rate within the smaller region to that of the larger, and let *s* equal the number of mutations shared by A00 and the reference sequence within the smaller region. With these, we constructed the estimator $\;{\hat{T}}_{\text{NM}}=s/\left(lr\mu \right)$. Similarly, let *L* equal the subset of the 8.8 Mb for which the A00 sequence had 3× or greater coverage (also ∼8.8 Mb), and let *S* equal the number of mutations unique to either the reference sequence or to A00 over the entire 8.8 Mb. We can estimate *T*_AR_ with ${\hat{T}}_{\text{AR}}=S/\left(2L\mu \right)$ and *α* with:

$$\hat{\alpha }=\left(1+\frac{{\hat{T}}_{\text{NM}}}{{\hat{T}}_{\text{AR}}}\right)=\left(1+\frac{2sL}{\hat{r}Sl}\right).$$

We estimated *r* by comparing the number of mutations unique to a single branch of the Y-chromosome tree of Karmin et al.,^15^ both within the full 8.8-Mb region and within the ∼80-kb subset. These numbers, 32,853 and 279 (238 under filter 4), respectively, correspond to a relative mutation rate of 0.93 (95% CI: 0.82–1.04) (0.84 under filter 4 [95% CI: 0.74–0.95]). Because selection has the strongest effect on lower frequency mutations, we also estimated *r* by using only shared variants, and this yielded nearly identical point estimates.

Finally, to construct a CI for *α*, we sampled values of *s* and *S* from Poisson distributions with means equal to the observed numbers of mutations, and we sampled *rl*/*L* as the ratio of two Poisson random variables with means equal to 279 (or 238) and 32,853, respectively.

### Functional Variation

We determined whether each mutation overlaps with annotated RefSeq genes and whether it overlaps with coding sequence (Figure 1, Data S5). For each coding SNV, we determined whether the mutation results in silent, missense, or nonsense mutations, but we did not consider frameshift mutations. For each coding non-synonymous mutation, we used the HumDiv model of PolyPhen-2 to evaluate ancestral-to-derived changes and MutationTaster to evaluate reference-to-alternative changes. We report findings from all sites for which these programs were able to make predictions.

## Results

With the chimpanzee Y chromosome as the outgroup, three tree topologies could have related the lineages of the Neandertal, haplogroup A00, and the human reference (Figure 1A). To identify which of the three was consistent with the data, the key question was which of the three possible pairs of sequences is the most closely related. Of 118,643 sites (Figure 1B, filter 1) for which we had Neandertal data and human-chimpanzee reference alignments,^18^ we identified 24 biallelic SNVs for which the Neandertal sequence shared the chimpanzee allele and differed from both A00 and the human reference. In contrast, the chimpanzee and A00 sequences shared just four SNVs not present in the other sequences, and the chimpanzee and human reference sequences shared zero. Taken together, these data strongly support the tree that places the Neandertal Y as the most distantly related to the others (Figure 1A, tree i). Two of the four variants that are inconsistent with this topology are known to segregate within modern humans and are therefore the result of recurrent mutations or contamination (Appendix A).

Upon elucidating the topology of the tree relating the Neandertal Y chromosome to those of modern humans, our next goal was to estimate the divergence time. We decomposed the TMRCA, *T*_NR_, into the sum of two intervals (Figure 2): the TMRCA of A00 and the reference, *T*_AR_, and the time between their common ancestor and the common ancestor of the Neandertal lineage and that of modern humans, *T*_NM_. To estimate *T*_NR_, we estimated *T*_AR_ and the ratio *α* ≡ *T*_NR_ ∕ *T*_AR_, taking care to consider uncertainty both in the mutation rate and in the expected number of mutations to construct a CI. Because the numbers of mutations that accumulate on the branches of the tree are conditionally independent of one another and are nearly uncorrelated with the estimator of *T*_AR_, we estimated *α* and *T*_AR_ independently (see Materials and Methods).

Leveraging data from an ∼45,000-year-old Siberian (Ust’-Ishim),^9^ we estimated that *T*_AR_ = 275 kya (95% CI: 241–305 kya), and we estimated *α* by using two approaches that yielded similar results. In our first approach, we simply used the number of mutations shared by A00 and the reference (branch a of Figure 2) and the number of mutations unique to each (branches d and e) to estimate the relative times between splits. This method is insensitive to mutation-rate variability across the chromosome and led us to estimate *α* = 2.14 (95% CI: 1.64–2.89). In the second approach, we made use of the greater amount of data available for the denominator of the ratio and adjusted for mutation rate heterogeneity across the chromosome to estimate *α* = 1.82 (95% CI: 1.40–2.32). Because the main source of uncertainty is the limited sequence coverage for the Neandertal lineage, the CIs from the two approaches overlap substantially, but we prefer the first method, as it is simpler and potentially less biased. In both cases, we disregarded the number of variants unique to the Neandertal sequence (branch f) because this branch is enriched for false positives as a result of low coverage, DNA damage, and sequencing errors.

Combining the parametric bootstrap CIs of *α* and *T*_AR_, we estimated *T*_NR_ = 588 kya (95% CI: 447–806 kya) with the first *α* estimate and *T*_NR_ = 499 kya (95% CI: 375–656 kya) with the second.

Finally, we examined the potential functional relevance of the 146 mutations that differed among the Neandertal, A00, and reference sequences (Data S5). These included 11 non-synonymous changes and one nonsense mutation (Table 1). PolyPhen-2^22^ predicted most missense mutations to have a benign effect, but it predicted possibly or probably damaging effects for Neandertal mutations in *PCDH11Y* (MIM: 400022) and *USP9Y* (MIM: 400005), for an A00 mutation in *ZFY* (MIM: 490000), and for a modern human mutation in *KDM5D* (MIM: 426000). The Neandertal nonsense mutation at codon 16 of *TMSB4Y* (MIM: 400017) might render its product non-functional, and MutationTaster^23^ predicts that it is probably deleterious.

## Discussion

**Figure 3:**
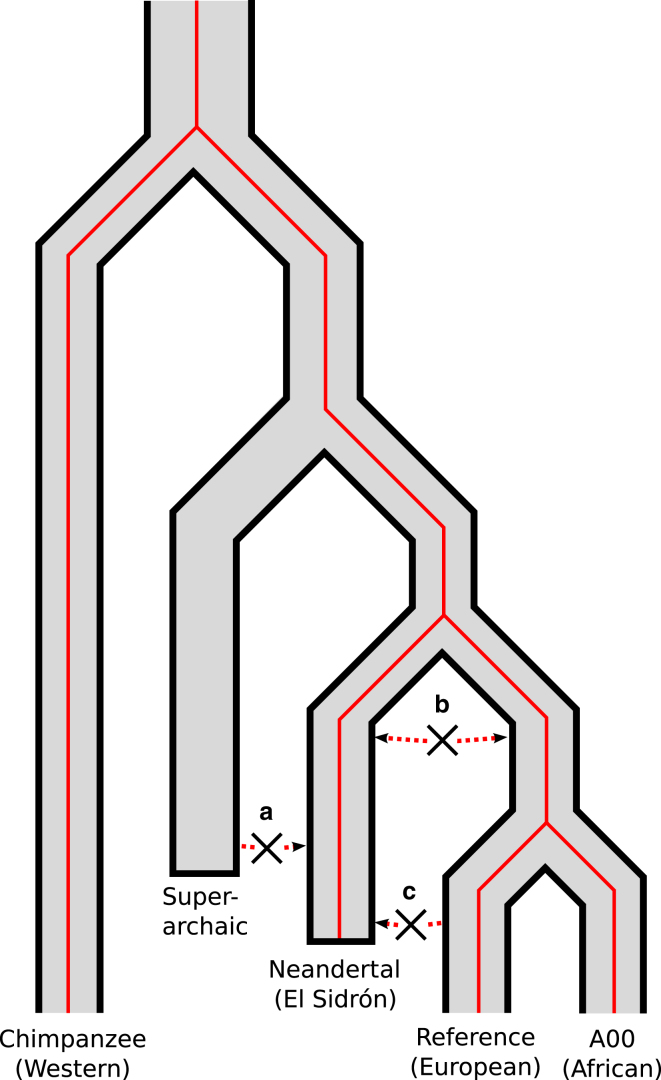
Relationship of Neandertal Y Chromosome to Those of Modern Humans The genealogy (red tree) can be parsimoniously explained as mirroring the population divergence (gray tree). We find no evidence for (a) a highly divergent super-archaic origin of the Neandertal Y chromosome, (b) ancient gene flow post-dating the population split, or (c) relatively recent introgression of a modern human Y chromosome into the Neandertal population.

We have estimated that the Neandertal Y chromosome from El Sidrón diverged from those of modern humans ∼590 kya, a value similar to TMRCA estimates for mtDNA sequences: 400 kya to 800 kya.^11^, ^12^ This time estimate and the genealogy we have inferred strongly support the notion that the most recent common ancestor of these Y chromosomes belonged to the population from which Neandertals and modern humans diverged, thereby refuting three alternative hypotheses. A priori, the Neandertal Y could have introgressed from a super-archaic population^5^ (Figure 3, scenario a), but this would have led to a far greater TMRCA estimate. Alternatively, it could have introgressed from the ancestors of modern humans after their divergence from Neandertals and prior to the most recent common ancestor of present-day Y chromosomes (scenario b) or from modern human populations subsequent to their migrations out of Africa (scenario c). We can also reject these hypotheses, as each requires a more recent split time.

The fact that the Neandertal Y-chromosome lineage we describe has never been observed in modern humans suggests that the lineage is most likely extinct. Although the Neandertal Y chromosome (and mtDNA) might have simply drifted out of the modern human gene pool,^24^ it is also possible that genetic incompatibilities contributed to their loss. In comparing the Neandertal lineage to those of modern humans, we identified four coding differences with predicted functional impacts, three missense and one nonsense (Table 1). Three mutations—within *PCDH11Y*, *USP9Y*, and *TMSB4Y*—are unique to the Neandertal lineage, and one, within *KMD5D*, is fixed in modern human sequences. The first gene, *PCDH11Y*, resides in the X-transposed region of the Y chromosome. Together with its X-chromosome homolog *PCDH11X*, it might play a role in brain lateralization and language development.^25^ The second gene, *USP9Y*, has been linked to ubiquitin-specific protease activity^26^ and might influence spermatogenesis.^27^ Expression of the third gene, *TMSB4Y*, might reduce cell proliferation in tumor cells, suggesting tumor suppressor function.^28^ Finally, the fourth gene, *KDM5D*, encodes a lysine-specific demethylase whose activity suppresses the invasiveness of some cancers.^29^

Polypeptides from several Y-chromosome genes act as male-specific minor histocompatibility (H-Y) antigens that can elicit a maternal immune response during gestation. Such effects could be important drivers of secondary recurrent miscarriages^30^ and might play a role in the fraternal birth order effect of male sexual orientation.^31^ Interestingly, all three genes with potentially functional missense differences between the Neandertal and modern humans sequences are H-Y genes, including *KDM5D*, the first H-Y gene characterized.^32^ It is tempting to speculate that some of these mutations might have led to genetic incompatibilities between modern humans and Neandertals and to the consequent loss of Neandertal Y chromosomes in modern human populations. Indeed, reduced fertility or viability of hybrid offspring with Neandertal Y chromosomes is fully consistent with Haldane’s rule, which states that “when in the [first generation] offspring of two different animal races one sex is absent, rare, or sterile, that sex is the [heterogametic] sex.”^33^

## Figures and Tables

**Table 1:** Protein-Changing Mutations

**Coordinate**	**Gene**	**Lineage**^1^	**Substitution**^2^	**Effect**	**Tool**	**Function**	**MIM No.**
2,844,774	*ZFY*	N	p.Val140Ala	B	P2	potential transcription factor	MIM: 490000
p.Val331Ala	B	P2
2,847,322	*ZFY*	A	p.Ile374Thr	B	P2	potential transcription factor	MIM: 490000
p.Ile488Thr	PrD	P2
p.Ile565Thr	B	P2
4,967,724	*PCDH11Y*^3^	N	p.Lys702Thr	B, B	P2, MT	protocadherin	MIM: 400022
5,605,569	*PCDH11Y*^3^	N	p.Ser1203Arg	PrD	P2	protocadherin	MIM: 400022
6,932,032	*TBL1Y*	N	p.Gly100Ala	B, B	P2, MT	–	MIM: 400033
14,832,610	*USP9Y*	N	p.Glu62Gly	PrD	P2	peptidase	MIM: 400005
14,832,620	*USP9Y*	R	p.Glu65Asp	B	P2	peptidase	MIM: 400005
14,838,553	*USP9Y*	N	p.Ala162Thr	B	P2	peptidase	MIM: 400005
15,816,262	*TMSB4Y*	N	p.Ser16^∗^	PrD	MT	actin sequestration	MIM: 400017
21,868,167	*KDM5D*	R, A	p.Arg1445Gln	B, B	P2, MT	demethylase	MIM: 426000
p.Arg1388Gln	B, B	P2, MT
p.Arg1476Gln	B, B	P2, MT
21,905,071	*KDM5D*	R, A	p.Ile69Val	PoD, B	P2, MT	demethylase	MIM: 426000
23,545,399	*PRORY*	A	p.Arg125Cys	–	–	–	–

Please see Data S5. Mutations variable among Neanderthal, A00, and the reference sequence, Data S6. Alignment (SAM) file with Neanderthal reads overlapping mutations in Table S3, Data S7. Neanderthal pileup lines for positions indicated in Table S3 for additional information on all mutations. Abbreviations are as follows: N, Neandertal; A, A00; R, reference; B, benign; PoD, possibly damaging; PrD, probably damaging; P2, PolyPhen-2 (ancestral to derived); MT, MutationTaster (reference to alternative).

1 Lineage(s) bearing the derived allele.

2 Multiple listings for a single coordinate reflect substitutions in different transcripts of the gene.

3 See Appendix B.

## Appendix A

### Incompatible and Recurrent Variants and *T*_AR_

In estimating *T*_AR_, we initially removed 46 sites with no chimpanzee alignment, 70 sites at which the chimpanzee base differed from both human bases, 13 sites at which the chimpanzee and reference sequences agreed (to the exclusion of the other two lineages), and 4 sites at which the chimpanzee and Ust’-Ishim lineages agreed (to the exclusion of the others). For the first two sets, A00 differs from the reference, so we could partition the mutations as either specific to the reference (branch d of Figure S2) or to the union of branches a and f (af). In both the set of 46 and the set of 70, the relative numbers of mutations assigned to branches d and af are consistent with those of the sites for which the chimpanzee data were conclusive (Fisher’s exact test p values: 0.82 and 0.13).

The 17 sites that are incompatible with the tree are principally due to recurrent and back mutations. Because the reference has accumulated more mutations than Ust’-Ishim since their common ancestor, it is expected that more incompatible sites unite A00 and Ust’-Ishim than unite the reference and A00. Indeed, 10 of the 13 mutations map to branches ancestral to the reference sequence (but not Ust’-Ishim) in the 1000 Genomes Project (see Web Resources). Likewise, one of the other four mutations could have recurred in the reference and A00. Our approach cannot detect mutations that occurred on both the lineage leading to A00 and on the lineage leading to K-M526; however, the expected number of such mutations is quite small.

Including the 116 mutations from the first two sets lowers the *T*_*AR*_ estimate from 287 kya (95% CI: 252–321 kya) to 284 kya (95% CI: 249–316 kya) and including the additional 11 mutations from the third and fourth sets lowers it further, to 275 kya (95% CI: 241–305 kya). However, this last estimate is most likely biased slightly downward due to the impossibility of observing mutations that recurred in the ancestors of A00 and of K-M526.

### Mutation Rate and TMRCA of K-M526

With the corrections described above, and assuming that the age of Ust’-Ishim is 45,000 years,^9^ we estimate the mutation rate in the analyzed region to be 0.78 × 10^−9^ mutations per bp per year (95% CI: 0.71–0.89 × 10^−9^ mutations per bp per year), and we estimate the TMRCA of K-M526 to be 48.1 kya (95% CI: 46.4–49.6 kya). The effective correction due to including the 127 mutations described above was small.

### Recurrent Mutations

Four mutations were inconsistent with tree ii in Figure 1A. Non-A0 lineages in the 1000 Genomes panel^34^ share the reference allele at coordinate 2,710,154, and individuals in haplogroups B through T share the reference allele at 23,558,260. The two others were at coordinates 9,386,241 and 15,024,530.

## Appendix B

The X-transposed region of the Y chromosome arose from the transposition of an ∼3.5-Mb stretch of the X chromosome at some point subsequent to the divergence of human and chimpanzee lineages.^20^ Due to sequence similarity of ∼99%, short-read mapping is often ambiguous in this region, but we were able to use accumulated sequence divergence to manually assess reads that mapped poorly to *PCDH11Y*.

The probably damaging functional mutation at GRCh37 coordinate 5,605,569 is flanked by two bases that differ between the X and Y chromosomes, at positions 5,605,520 and 5,605,622. Seven sequencing reads overlapped the putative functional mutation and at least one of these two Y-specific bases, and each supported a derived allele call for the Neandertal lineage. Thus, despite the fact that just one of the seven reads mapped with high quality, we had sufficient evidence to call the derived genotype. Furthermore, the only read that carried the ancestral allele at the functional site, and overlapped one of the two diagnostic sites, bore the X-specific base.

## Web Resources

The URLs for data presented herein are as follows:

1000 Genomes Project Y Chromosome Supporting Data, ftp://ftp.1000genomes.ebi.ac.uk/vol1/ftp/release/20130502/supporting/chrY/
MutationTaster, http://www.mutationtaster.org/
OMIM, http://www.omim.org/
PolyPhen-2, http://genetics.bwh.harvard.edu/pph2/
UCSC Genome Browser, http://genome.ucsc.edu

## Supplemental Data

Document S1. Figures S1 and S2

Data S1. Regions passing Filter 1

Data S2. Regions passing Filter 2

Data S3. Regions passing Filter 3

Data S4. Regions passing Filter 4

Data S5. Mutations variable among Neanderthal, A00, and the reference sequence

Data S6. Alignment (SAM) file with Neanderthal reads overlapping mutations in Table S3

Data S7. Neanderthal pileup lines for positions indicated in Table S3

Document S2. Spanish Translation: La Divergencia de Los Cromosomas Y de Neandertal y de los Humanos Modernos

Document S3. Article plus Supplemental Data
