# Supplementary material for: The Divergence of Neandertal and Modern Human Y Chromosomes
Source: Am J Hum Genet. 2016 Apr 7;98(4):728–34. doi: 10.1016/j.ajhg.2016.02.023 (PMC4833433; doi:10.1016/j.ajhg.2016.02.023)
Supplement: Document S1. Figures S1 and S2 [file mmc1.pdf]

The American Journal of Human Genetics, Volume *98*

Supplemental Data

The Divergence of Neandertal and Modern Human Y Chromosomes

Fernando L. Mendez, G. David Poznik, Sergi Castellano, and Carlos D. Bustamante

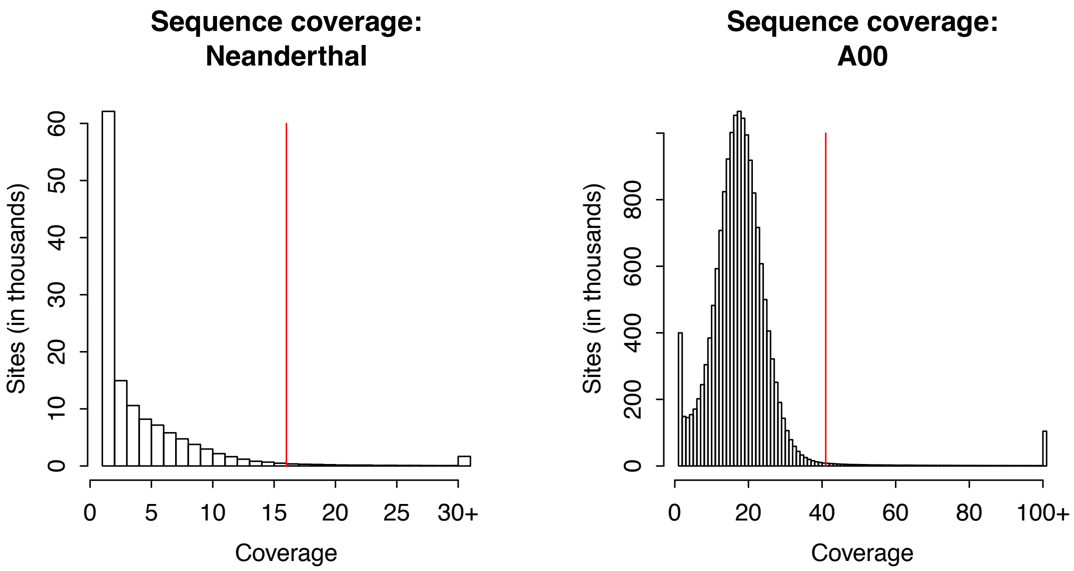

Figure S1. Sequencing coverage. Red lines indicate values exceeding the sample means by 5 times their square root. We discarded sites whose sequence coverages were greater than these values.

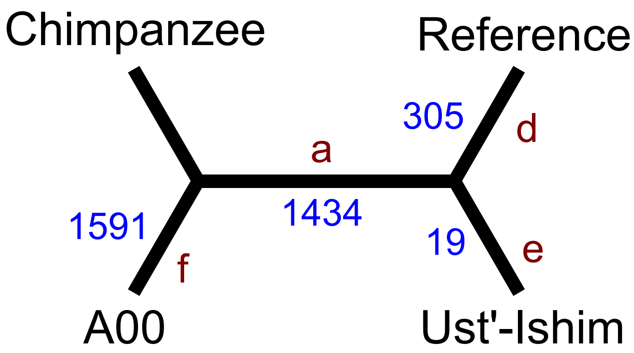

Figure S2. Estimating T_{AR}. Blue numbers indicate counts of mutations within ~7.83 Mb of overlapping sequence.
