# Supplementary material for: The Divergence of Neandertal and Modern Human Y Chromosomes
Source: Am J Hum Genet. 2016 Apr 7;98(4):728–34. doi: 10.1016/j.ajhg.2016.02.023 (PMC4833433; doi:10.1016/j.ajhg.2016.02.023)
Supplement: Document S2. Spanish Translation: La Divergencia de Los Cromosomas Y de Neandertal y de los Humanos Modernos [file mmc9.pdf]

La divergencia de los cromosomas Y de Neandertal y de los humanos modernos

Fernando L. Mendez[1,*], G. David Poznik[1,2], Sergi Castellano[3], Carlos D. Bustamante[1,4,*]

[1]Department of Genetics, Stanford University, Stanford, CA 94305, USA
[2]Program in Biomedical Informatics, Stanford University, Stanford, CA 94305, USA
[3]Department of Evolutionary Genetics, Max Planck Institute for Evolutionary Anthropology, Leipzig 04103, Germany
[4]Department of Biomedical Data Science, Stanford University, Stanford, CA 94305, USA
*Correspondence: flmendez@stanford.edu (F.L.M.), cdbustam@stanford.edu (C.D.B.)

Resumen

La secuenciación de los genomas de homínidos extintos ha redefinido nuestra comprensión de los orígenes de los humanos modernos. Analizamos ~ 120 kb de una captura de exoma del ADN del cromosoma Y de un individuo neandertal de El Sidrón, España. Investigamos su divergencia con la de secuencias ortólogas de chimpancé y de humanos modernos. Encontramos un fuerte apoyo a un modelo que coloca el linaje neandertal como grupo externo al de los cromosomas Y de los humanos modernos, incluyendo A00, un haplogrupo basal muy divergente. Estimamos que el tiempo desde el ancestro común más reciente de los cromosomas Y de neandertal y de los humanos modernos es de aproximadamente 588 mil años (95% IC: 447–806 mil años). Este es ~2.1 (95% IC: 1.7-2.9) veces más largo que aquel del ancestro común entre A00 y los otros linajes humanos existentes. Esta estimación sugiere que la divergencia del cromosoma Y refleja la divergencia entre las poblaciones ancestrales a los neandertales y a los humanos modernos, y refuta escenarios alternativos de un origen relativamente reciente o superarcaico de los cromosomas Y de neandertales. El hecho de que el cromosoma Y de neandertal que describimos nunca haya sido observado en los humanos modernos, sugiere que este linaje probablemente esté extinto. En este estudio, identificamos diferencias en algunas de las proteínas codificadas por los cromosomas Y del neandertal y de los humanos modernos, incluyendo cambios potencialmente perjudiciales en los genes *PCDH11Y*, *TMSB4Y*, *USP9Y* y *KDM5D*. Tres de estos cambios producen sustituciones en las cadenas aminoacídicas, y estos tres genes producen antígenos menores de histocompatibilidad masculino-específicos (H-Y). Por ejemplo, se cree que antígenos derivados de *KDM5D* provocan una respuesta inmune materna durante la gestación. Es posible que incompatibilidades en uno o más de estos genes hayan jugado un papel en el aislamiento reproductivo de los dos grupos.

Introducción

Uno de los objetivos centrales de la Genética de Poblaciones Humanas y la Paleoantropología es dilucidar las relaciones entre las poblaciones antiguas. Previo a la aparición de los humanos anatómicamente modernos en el Pleistoceno Medio hace ~ 200 mil años [1], los humanos arcaicos vivían en África, Europa y Asia en poblaciones

altamente diferenciadas. Las poblaciones humanas modernas que se expandieron fuera de África durante el Pleistoceno Superior recibieron una modesta contribución genética de al menos dos grupos de homínidos arcaicos: los neandertales y los denisovas [2-5]. Especialmente a la luz de las hipotéticas incompatibilidades genéticas entre los neandertales y los humanos modernos [6], es importante el caracterizar la diferenciación entre sus poblaciones ancestrales e investigar posibles barreras al flujo genético.

Cuando las poblaciones divergen entre sí, cada una conserva una parte de la variación existente en la población ancestral. Consecuentemente, los tiempos de divergencia de las secuencias suelen superar a los tiempos de divergencia de las poblaciones, y este efecto es más pronunciado cuando el tamaño efectivo de la población ancestral era grande. En los seres humanos, una fracción importante de la diversidad genética se debe a polimorfismos antiguos que surgieron mucho antes de la aparición de rasgos anatómicos modernos. Como resultado, los haplotipos de los neandertal y de los modernos son, a menudo, no más divergentes de lo que lo son entre sí las secuencias humanas modernas,[2] y este hecho complica la búsqueda de segmentos genómicos de introgresión. Sin embargo, dos características facilitan el descubrimiento de estos últimos [6,7]. En primer lugar, debido a los bajos niveles de polimorfismo entre los neandertales, [5] las secuencias de introgresión son a menudo muy similares a las de las de referencia del neandertal. En segundo lugar, estas regiones presentan un desequilibrio de ligamiento elevado debido a que la mezcla ocurrió en fecha relativamente reciente, hace aproximadamente 50 000 años [8-10]. Aunque se han identificado secuencias de introgresión neandertal en autosomas y en el cromosoma X de humanos modernos, ninguna secuencia de genoma mitocondrial (ADNmt) de origen neandertal se ha reportado en los seres humanos modernos, y las secuencias del cromosoma Y de neandertales aún no han sido caracterizadas.

Debido a que los tamaños efectivos de población de los *loci* heredados de forma uniparental son mucho menores que aquellos de los *loci* autosómicos o ligados al cromosoma X, las diferencias esperadas entre tiempos de divergencia de la secuencia y de las poblaciones son más pequeñas. Por lo tanto, el estudio de estos *loci* puede ayudar a determinar un límite superior para el tiempo transcurrido desde la última vez que las poblaciones intercambiaron material genético. Hasta la fecha, se han secuenciado los genomas completos de cinco individuos neandertales con 0.1x de cobertura o mayor [2,5], pero todos eran hembras. También hay disponibles secuencias completas del ADNmt de ocho individuos de España, Alemania, Croacia y Rusia [11,12], pero la relación entre el cromosoma Y neandertal y el de humanos modernos se desconoce aún.

En este trabajo, analizamos ~ 120 kb de la captura de exoma de la secuencia del cromosoma Y de un neandertal macho de aproximadamente 49.000 años (^{14}C no calibrado)[13], proveniente de El Sidrón, España [14]. Comparamos la secuencia con las de referencia para humanos y chimpancés, y con las secuencias de dos individuos Mbo [15] que llevan el haplogrupo A00, el grupo con la ramificación más antigua conocida [16]. Identificamos la relación entre los cromosomas Y del neandertal y del hombre moderno, y estimamos el tiempo de su ancestro común más reciente (TACMR). También examinamos las diferencias codificantes y exploramos su potencial importancia para el aislamiento reproductivo.

Materiales y Métodos

Datos de secuencia y procesamiento

Usamos la secuencia del cromosoma Y de la captura de exoma de un neandertal de El Sidrón, España [14] y descargamos las secuencias completas de dos cromosomas Y de A00 [15]. Los datos del individuo neandertal incluyeron secuencias codificantes, no codificantes y secuencias no seleccionadas especialmente por la captura; las secuencias de estos tres tipos fueron mapeadas contra la referencia hg19. Como las secuencias de A00 tenían una relación muy estrecha [15,16], las combinamos para incrementar la cobertura. Usamos SAMtools mpileup (versión 1.1) [17] para determinar las secuencias tanto de neandertal como de A00, especificando opciones de entrada para contar pares de lectura anómalos (-A), recalcular la calidades de lectura (-E), y establecer los umbrales de la calidad de lectura (-Q 17) y la calidad del mapeo (-q 20).

Luego identificamos las regiones solapadas y excluimos coordenadas para las cuales la cobertura era inusualmente alta, filtrando los sitios con cobertura mayor a la media más 5 veces su raíz cuadrada (**Figura S1**). Bajo un modelo de Poisson, este punto de corte provocaría la pérdida de menos de uno de cada 10 000 sitios genuinos. Por último, eliminamos los sitios que presentan inconsistencias en las secuencias producidas por los fragmentos que las cubren. Descartamos aquellos sitios en los que más de dos fragmentos indican un alelo distinto del consenso y aquellos para los cuales más de un tercio de los nucleótidos observados no coinciden con el consenso. Este filtro debería reducir al mínimo los efectos de daño en el ADN *post mortem* y de la contaminación moderna.

Usando el archivo de blastz chrY.hg19.panTro4.net.axt.gz [18], identificamos el subconjunto de regiones dentro de las cuales las secuencias humanas se alinean con la de referencia de chimpancé. Esto arrojó un total de 118 643 pares de bases (pb). En lo que sigue, nos referiremos a este conjunto de sitios como 'Filtro 1'. También identificamos un segundo conjunto de regiones, más restringido, con un total de 100 324 pares de bases, 'Filtro 2', al requerir también que el alineamiento correspondiera al cromosoma Y, y no a otro cromosoma en chimpancé (**Tablas S1a** y **S1b**).

Para cada posición dentro de estas regiones, determinamos si el neandertal, A00, o ambos, diferían de la secuencia de referencia humana. A continuación, con el fin de asignar la mutación a la correspondiente rama del árbol que relaciona las cuatro secuencias (**Figura 1a**), utilizamos el alelo correspondiente de chimpancé como representante del estado ancestral. De este modo, se descartaron cinco sitios: dos en los cuales el cromosoma de chimpancé tiene un tercer alelo, uno en el que el cromosoma de chimpancé tiene una deleción, y dos que eran específicos para el A00 pero que estaban apoyados solamente por una única secuencia. La exclusión de estos sitios tuvo un efecto pequeño en nuestro análisis.

La estimación de TACMR

Para estimar el TACMR de los cromosomas Y del neandertal y del humano moderno (T_{NR}), descompusimos esta cantidad (**Figura 2**) como la suma de los TACMR de los humanos modernos (T_{AR}) y el tiempo que separa al último ancestro común de los

humanos modernos de su ancestro común con el linaje neandertal:

$$T_{NR} = T_{AR} + T_{NM} = \alpha T_{AR}$$
$$\alpha \equiv \left(1 + \frac{T_{NM}}{T_{AR}}\right)$$

A continuación, estimamos T_{AR} y usamos dos métodos para estimar α.

Para estimar T_{AR}, utilizamos los datos de secuencia de la muestra antigua de Ust-Ishim [9], previamente aplicando los filtros descriptos para la secuencias de A00. Para reducir el impacto potencial del daño *post mortem* en el ADN, limitamos el análisis a las coordenadas cubiertas por al menos tres lecturas de secuenciación. Restringimos aún más el análisis al subconjunto de las regiones de Poznik et al. (2013) [19] en los que la secuencia de referencia humana se basa en clones de BAC derivados del individuo RP-11 [20], quien porta un cromosoma Y del haplogrupo R1b. Esto deja ~7,83 Mb de secuencia dentro de los cuales asignar variantes a las ramas apropiadas (**Figura S2**, **Apéndice A**). Usando la edad conocida del individuo Ust'-Ishim y el procedimiento de optimización con restricciones descripto en Rasmussen et al. (2014) [21], obtuvimos estimaciones de bootstrap paramétrico para T_{AR}, así como para la tasa de mutación y la TACMR del haplogrupo K-M526 (**Apéndice A**). En pocas palabras, tomamos muestras del proceso que generó el árbol observado (**Figura S2**), simulando el número de variantes nucleotídicas (SNVs, por sus siglas en inglés) en cada rama a partir de una distribución Poisson con media igual al número observado de mutaciones. A continuación, maximizamos la probabilidad de replicar cada árbol con respecto a T_{AR}, la tasa de mutación y el TACMR de K-M526 para así obtener muestras de bootstrap de estos tres parámetros.

En nuestro primer enfoque para estimar α, utilizamos el número relativo de mutaciones asignadas a las ramas a, d, y e (**Figura 1**), asignando los cuatro sitios que no se ajustaban a la topología de consenso al A00 o al linaje de referencia, según fuese el caso (**Apéndice A**). La proporción del tiempo representado para la rama a es:

$$\frac{T_{\mathrm{a}}}{T_{\mathrm{a}} + T_{\mathrm{b}} + T_{\mathrm{e}}} = \frac{T_{\mathrm{NM}}}{T_{\mathrm{NM}} + 2T_{\mathrm{AR}}} = \frac{(\alpha - 1)T_{\mathrm{AR}}}{(\alpha - 1)T_{\mathrm{AR}} + 2T_{\mathrm{AR}}} = \frac{\alpha - 1}{\alpha + 1}$$

Por lo tanto, suponiendo que la tasa de mutación es homogénea en el tiempo, el número de mutaciones del ramal a tiene una distribución binomial con parámetros $p = (\alpha - 1)/(\alpha + 1)$ y n igual al número total de mutaciones. La estimación de p a partir de los datos conduce directamente a una estimación puntual y al intervalo de confianza para α. Este primer método tiene la atractiva propiedad que ser independiente tanto de la tasa de mutación, como de los valores absolutos de los tiempos. Sin embargo, el error de estimación puede ser sub-óptimo debido a la presencia de incertidumbre tanto en el numerador y como en el denominador.

En el segundo método, estimamos α a través de la relación $T_{\mathrm{NM}}/T_{\mathrm{AR}}$, teniendo en cuenta que la estimación de T_{AR} tendrá mayor certeza que la de T_{NM}. Para estimar T_{NM}, limitamos nuestra atención a las secuencias que se superponen con las ~8,8 Mb analizadas por Karmin et al. [15], quedando 80 420 pb (Filtro 3), o 75 596 pb (Filtro 4) cuando restringimos nuestro análisis a aquellos sitios que pasaron el Filtro 2 (**Figura 1**,

Tablas S2a y S2b). Sean l la longitud total de la secuencia en cuestión (e. g., 80,42 kb), μ la tasa de mutación sobre los 8,8 Mb completos, r la relación de la tasa de mutación dentro de la región más pequeña y la más grande y s el número de mutaciones compartidas por A00 y la secuencia de referencia dentro de la región más pequeña. Con éstos, construimos el estimador $\hat{T}_{\mathrm{NM}} = s/(lr\mu)$. De igual modo, sean L el subconjunto de los 8,8 Mb para el cual la secuencia del A00 tiene 3 × o mayor cobertura (también ~8,8 Mb), y S el número de mutaciones únicas, ya sea a la secuencia de referencia o al A00, sobre la totalidad de las 8,8 Mb. Podemos estimar T_{AR} con $\hat{T}_{\mathrm{AR}} = S/(2L\mu)$ y α con:

$$\hat{\alpha} = \left(1 + \frac{\hat{T}_{\mathrm{NM}}}{\hat{T}_{\mathrm{AR}}}\right) = \left(1 + \frac{2sL}{\hat{r}Sl}\right)$$

Estimamos r comparando el número de mutaciones que ocurren en una única rama del árbol del cromosoma Y de Karmin et al. [15], tanto dentro de toda la región de los 8,8 Mb como dentro del subconjunto de ~ 80 kb. Estos números, 32 853 y 279 (238 para el filtro 4), respectivamente, corresponden a una tasa de mutación relativa de 0,93 (95% IC: 0,82-1,04) (0,84 [95% IC: 0,74-0,95] para el filtro 4). Debido a que el efecto de la selección es más fuerte en las mutaciones de frecuencia más baja, también estimamos r usando solamente variantes compartidas, resultando en estimaciones puntuales casi idénticas.

Finalmente, para construir un intervalo de confianza para α, muestreamos valores de s y S a partir de distribuciones Poisson con medias iguales al número de mutaciones observadas y muestreamos rl/L como la razón entre dos variables aleatorias Poisson con medias iguales a 279 (238) y 32 853, respectivamente.

Variación funcional

Determinamos si cada mutación se solapa con genes anotados en RefSeq y si se superponen con la secuencia codificante (**Figura 1**, **Tabla S3**). Para cada SNVs codificante, determinamos si la mutación es silenciosa, resulta en cambios de la secuencia aminoacídica, o si son mutaciones terminadoras, pero no consideramos mutaciones que afecten el marco de lectura. Para cada mutación no sinónima, usamos el modelo de HumDiv de PolyPhen-2 para evaluar los cambios yendo de ancestral a derivado, y MutationTaster para evaluar los cambios yendo de la referencia a la variante alternativa. Reportamos los resultados de todos los sitios en los que estos programas fueron capaces de hacer predicciones.

Resultados

Con el cromosoma Y del chimpancé como grupo externo, tres árboles distintos podrían haber relacionado los linajes del neandertal, el haplogrupo A00, y la referencia humana (**Figura 1a**). Para identificar cuál de los tres era consistente con los datos, la pregunta clave era cuál de los tres posibles pares de secuencias estaban más estrechamente relacionados. De los 118 643 sitios (**Figura 1b**, Filtro 1) para los que teníamos datos de neandertal y los alineamientos de referencia entre humanos y chimpancés [18], identificamos 24 SNVs bialélicos para los que la secuencia de neandertal

comparte el alelo de chimpancé y difiere tanto del A00 como de la humana de referencia. Por otro lado, las secuencias de chimpancé y A00 comparten sólo cuatro SNVs que no están presentes en las otras secuencias, y las secuencias de chimpancé y humano de referencia no comparten ninguna. En conjunto, estos datos apoyan firmemente el árbol que coloca al Y de neandertal como el más distantemente relacionado a los otros (**Figura 1a**, árbol *i*). Se sabe que dos de las cuatro variantes que son inconsistentes con esta topología segregan entre los humanos modernos y por lo tanto su presencia es el resultado de mutaciones recurrentes o contaminación (**Apéndice A**).

Tras aclarar la topología del árbol que relaciona a los cromosoma Y del neandertal y del humano moderno, nuestro siguiente objetivo fue el estimar el tiempo de divergencia. Descompusimos la TACMR, T_{NR}, como la suma de dos intervalos (**Figura 2**): la TACMR de A00 y la referencia, T_{AR} y el tiempo entre su ancestro común y el ancestro común con el linaje neandertal, T_{NM}. Para estimar T_{NR}, estimamos T_{AR} y la relación $\alpha \equiv T_{NR}/T_{AR}$, teniendo cuidado —al construir un intervalo de confianza— de considerar tanto la incertidumbre en la tasa de mutación como en el número esperado de mutaciones. Debido a que los números de mutaciones que se acumulan en las ramas del árbol son condicionalmente independientes uno de otro y son casi no correlacionadas con el estimador de T_{AR}, estimamos α y T_{AR} de manera independiente (**Materiales y Métodos**).

Aprovechando los datos de un siberiano de aproximadamente 45 000 años de antigüedad (Ust'-Ishim) [9], estimamos que T_{AR} = 275 mil años (95% IC: 241-305 mil años) y estimamos α usando dos enfoques que arrojaron resultados similares. En nuestro primer enfoque, simplemente empleamos el número de mutaciones compartidas por A00 y la referencia (rama a de la **Figura 2**) y el número de mutaciones únicas para cada una (ramas d y e) para estimar los tiempos relativos entre divisiones. Este método es insensible a la variabilidad en la tasa de mutación a lo largo del cromosoma y nos llevó a estimar α = 2,14 (95% IC: 1,64 – 2,89). En el segundo enfoque, aprovechamos la mayor cantidad de datos disponibles para el denominador de la razón y ajustamos la estimación teniendo en cuenta lo heterogéneo de la tasa de mutación a lo largo del cromosoma para estimar α = 1,82 (95% IC: 1,40 – 2,32). Dado que la principal fuente de incertidumbre es la limitación en la cobertura de la secuencia del linaje neandertal, los intervalos de confianza resultantes de los dos enfoques se superponen sustancialmente; aun así preferimos el primer método, ya que es más sencillo y potencialmente menos sesgado. En ambos casos, prescindimos de la cantidad de variantes únicas para la secuencia de neandertal (rama f) ya que esta rama está enriquecida con falsos positivos debido a la baja cobertura, el daño en el ADN y los errores de secuenciación.

Combinando los intervalos de confianza del bootstrap paramétrico de α y T_{AR}, estimamos T_{NR} = 588 mil años (95% IC: 447-806 mil años) al usar la primera estimación para α y T_{NR} = 499 mil años (95% IC: 375-656 mil años) al usar la segunda.

Por último, analizamos la potencial relevancia funcional de las 146 mutaciones que difieren entre los neandertal, A00, y secuencias de referencia (**Tabla S3**). Estas incluyeron 11 cambios no sinónimos y 1 mutación sin sentido (**Tabla 1**). PolyPhen-2 [22] predijo que la mayoría de las mutaciones que afectan la secuencia aminoacídica tienen un efecto benigno, pero también predijo efectos posible o probablemente perjudiciales asociados a las mutaciones de neandertal en *PCDH11Y* (MIM: 400022) y *USP9Y* (MIM: 400005), una mutación de A00 en *ZFY* (MIM: 490000) y una mutación en humanos modernos en *KDM5D* (MIM: 426000). La mutación sin sentido en el codón 16 de

TMSB4Y (MIM: 400017) en neandertal puede provocar que su producto sea no funcional, y MutationTaster [23] predice que probablemente sea perjudicial.

Discusión

Hemos estimado que el cromosoma Y del neandertal de El Sidrón divergió del de los humanos modernos hace aproximadamente 590 mil años, un valor similar al TACMR estimado para las secuencias de ADNmt: 400 mil años a 800 mil años [11,12]. Esta estimación y la genealogía que hemos inferido, apoyan fuertemente la noción de que el ancestro común más reciente de los cromosomas Y pertenecía a la población de la cual divergieron los neandertales y los humanos modernos, rebatiendo así tres hipótesis alternativas. *A priori*, el Y de neandertal podría originarse en una introgresión de una población "super-arcaica" [5] (**Figura 3**, escenario *a*), pero esto habría dado lugar a una estimación de TACMR mucho mayor. Como alternativa, podría haber pasado de los ancestros de los humanos modernos, con posterioridad a su divergencia de los neandertales y antes del ancestro común más reciente de los actuales cromosomas Y (escenario *b*), o de las poblaciones humanas modernas con posterioridad a su migración fuera de África (escenario *c*). También podemos rechazar estas hipótesis, ya que requieren un tiempo divergencia más reciente.

El hecho de que el cromosoma Y del neandertal que hemos descripto nunca haya sido observado en los seres humanos modernos, sugiere que este linaje está probablemente extinto. Mientras que el cromosoma Y del neandertal (y el ADNmt) puede haberse extinguido del pool de genes de humanos modernos debido a deriva genética [24], también es posible que incompatibilidades genéticas contribuyeran a su pérdida. Al comparar el linaje neandertal con los de los humanos modernos, identificamos cuatro diferencias codificantes con predicciones de impacto funcionales, tres mutaciones que producen cambios en la secuencia aminoacídica y una terminadora (**Tabla 1**). Tres mutaciones, en *PCDH11Y*, *USP9Y*, y *TMSB4Y* están presentes solo en el linaje neandertal, y una, en *KMD5D*, está fija en las secuencias de humanos modernos. El primer gen, *PCDH11Y*, se ubica en la región X transpuesta del cromosoma Y. Junto con su homólogo del cromosoma X, *PCDH11X*, puede desempeñar un papel en la lateralización del cerebro y en el desarrollo del lenguaje [25]. El segundo gen, *USP9Y*, se ha relacionado con la actividad de proteasa específica de ubiquitina [26] y podría influir en la espermatogénesis [27]. La expresión del tercer gen, *TMSB4Y*, podría llegar a reducir la proliferación celular en las células tumorales, sugiriendo una función supresora de tumores [28]. Por último, el cuarto gen, *KDM5D*, codifica una demetilasa lisina-específica cuya actividad suprime la invasividad de algunos tipos de cáncer [29].

Los polipéptidos de varios genes del cromosoma Y actúan como antígenos menores de histocompatibilidad masculino-específicos (H-Y), que podrían provocar una respuesta inmune materna durante la gestación. Tales efectos podrían ser importantes impulsores de abortos secundarios recurrentes [30] y podrían desempeñar un papel en el "efecto del hermano mayor" de la orientación sexual masculina [31]. Curiosamente, los tres genes con diferencias de cambio de aminoácidos potencialmente funcional entre las secuencias de neandertal y las de humanos modernos son genes H-Y, incluyendo *KDM5D*, el primer gen H-Y caracterizado [32]. Es tentador especular que algunas de estas mutaciones podrían haber dado lugar a incompatibilidades genéticas entre los humanos

modernos y los neandertales y a la consiguiente pérdida de los cromosomas Y de neandertal en las poblaciones modernas. De hecho, la reducción en la fertilidad o en la viabilidad de la descendencia híbrida con cromosomas neandertal Y es plenamente compatible con la regla de Haldane, que establece que ‘cuando en la descendencia F_1 [primera generación de la cruza] de dos razas diferentes de animales un sexo está ausente, es raro o estéril, ese sexo es el heterocigota [heterogamético]’. [33]

Apéndice A

Variantes recurrentes e incompatibles y T_{AR}

En la estimación de T_{AR}, inicialmente eliminamos 46 sitios para los que no había alineamiento con chimpancé, 70 sitios en los cuales la base de chimpancé discrepó de ambas bases humanas, 13 sitios en los que las secuencias de chimpancé y de referencia concordaron (con exclusión de los otros dos linajes), y 4 sitios en los que los linajes de chimpancé y de Ust-Ishim coincidieron (con exclusión de los demás). Para los dos primeros conjuntos, A00 difiere de la referencia, por lo que sería posible particionar el conjunto de mutaciones de acuerdo a si son específicas a la referencia (rama d de **Figura S2**) o a la unión de las ramas a y f (af). Tanto en el grupo de 46 como en el de 70, el número relativo de mutaciones asignadas a las ramas d y af son consistentes con los de los sitios en los que los datos de los chimpancés eran concluyentes (los valores de *p* del test exacto de Fisher son: 0,82 y 0,13).

Los 17 sitios que son incompatibles con el árbol, obedecen principalmente a mutaciones recurrentes y reversiones. Dado que desde su ancestro común la referencia ha acumulado más mutaciones que en Ust'- Ishim, es esperable que más sitios incompatibles unan al A00 y al Ust-Ishim que los que unen a la referencia y al A00. De hecho, 10 de las 13 de mutaciones mapean en ramas ancestrales a la secuencia de referencia (pero no a Ust-Ishim) en el del *Proyecto 1000 Genomas*. Del mismo modo, una de las otras 4 mutaciones podrían haber ocurrido en la referencia y en el A00. Nuestro enfoque no puede detectar las mutaciones que ocurrieran en los linajes ancestrales a A00 y a K-M526; sin embargo, el número esperado de tales mutaciones es bastante pequeño.

El incluir las 116 mutaciones de los dos primeros conjuntos disminuye la estimación de T_{AR} de 287 mil años (95% IC: 252-321 mil años) a 284 mil años (95% IC: 249-316 mil años) y el incluir las 11 mutaciones adicionales del tercer y cuarto conjunto, la reduce aún más a 275 mil años (95% IC: 241-305 mil años). Sin embargo, esta última estimación probablemente sea ligeramente sesgada a la baja debido a la imposibilidad de observar las mutaciones que se produjeron en los antepasados del A00 y del K-M526.

Tasa de mutación y TACMR de K-M526.

Con las correcciones descriptas anteriormente, y suponiendo que la edad de Ust-Ishim es de 45 mil años [9], estimamos que la tasa de mutación en la región analizada es: 0,78 x 10^{-9} mutaciones/pb/año (95% IC:0,71-0,89 x 10^{-9} mutaciones/pb/año) y estimamos que la TACMR de K-M526 es de 48.1 mil años (95% IC: 46,4-49,6 mil años). La corrección efectiva, debido a las 127 mutaciones incluidas anteriormente, fue pequeña.

Mutaciones recurrentes

Cuatro mutaciones eran incompatibles con el árbol *ii* en la **Figura 1a**. Los linajes en el panel de 1000 genomas que no son A0 comparten el alelo de referencia en la coordenadas 2,710,154, y los individuos en haplogrupos del B hasta el T comparten el alelo de referencia en 23 558 260. Las otras dos mutaciones estaban en las coordenadas 9 386 241 y 15 024 530.

Apéndice B

La región 'X transpuesta' del cromosoma Y surgió de la transposición de un segmento de ~3,5 Mb del cromosoma X en algún momento posterior a la divergencia entre los linajes humano y de chimpancé [20]. Debido a la similitud de secuencias de ~ 99%, el mapeo de secuencias breves es a menudo ambiguo en esta región, pero fuimos capaces de usar algunas de las diferencias acumuladas en la secuencia para evaluar manualmente las secuencias de fragmentos que mapeaban deficientemente al gen *PCDH11Y*.

La mutación probablemente dañina a nivel funcional que en GRCh37 tiene coordenadas 5 605 569, está flanqueada por dos bases que difieren entre los cromosomas X e Y, en las posiciones 5 605 520 y 5 605 622. Siete fragmentos se superpusieron a la mutación putativa funcional y a al menos una de estas dos bases específicas del Y, y cada uno apoyó la presencia del alelo derivado para el linaje neandertal. Así, a pesar del hecho de que sólo una de los siete secuencias está mapeada con alta calidad, tuvimos suficientes pruebas para decidir la presencia del genotipo derivado. Además, la única secuencia que llevaba el alelo ancestral en el sitio funcional, se superponía a uno de los dos sitios de diagnóstico, y llevaba la base-X específica.

Datos suplementarios

Los datos suplementarios incluyen dos figuras, tres tablas, y dos archivos de datos.

Agradecimientos

Ver versión en inglés para apoyo financiero. Agradecemos a Bence Viola por ofrecer sus conocimientos y a Chris Gignoux por sugerencias y comentarios útiles.

Recursos Web

Los URLs para los datos presentados aquí son los siguientes:
1000 Genomes Project Y Chromosome Supporting Data,
ftp://ftp.1000genomes.ebi.ac.uk/vol1/ftp/release/20130502/supporting/chrY/
Mutation Taster, http://www.mutationtaster.org/
Online Mendelian Inheritance in Man (OMIM), http://www.omim.org
Polyphen2, http://genetics.bwh.harvard.edu/pph2/index.shtml
UCSC Genome Browser, http://genome.ucsc.edu/

Referencias

1. McDougall, I., Brown, F.H., and Fleagle, J.G. (2005). Stratigraphic placement and age of modern humans from Kibish, Ethiopia. Nature *433*, 733–736.

2. Green, R.E., Krause, J., Briggs, A.W., Maricic, T., Stenzel, U., Kircher, M., Patterson, N., Li, H., Zhai, W., Fritz, M.H.-Y., et al. (2010). A draft sequence of the Neandertal genome. Science *328*, 710–722.

3. Reich, D., Green, R.E., Kircher, M., Krause, J., Patterson, N., Durand, E.Y., Viola, B., Briggs, A.W., Stenzel, U., Johnson, P.L.F., et al. (2010). Genetic history of an archaic hominin group from Denisova Cave in Siberia. Nature *468*, 1053–1060.

4. Meyer, M., Kircher, M., Gansauge, M.-T., Li, H., Racimo, F., Mallick, S., Schraiber, J.G., Jay, F., Prüfer, K., de Filippo, C., et al. (2012). A High-Coverage Genome Sequence from an Archaic Denisovan Individual. Science *338*, 222–226.

5. Prüfer, K., Racimo, F., Patterson, N., Jay, F., Sankararaman, S., Sawyer, S., Heinze, A., Renaud, G., Sudmant, P.H., de Filippo, C., et al. (2014). The complete genome sequence of a Neanderthal from the Altai Mountains. Nature *505*, 43–49.

6. Sankararaman, S., Mallick, S., Dannemann, M., Prüfer, K., Kelso, J., Pääbo, S., Patterson, N., and Reich, D. (2014). The genomic landscape of Neanderthal ancestry in present-day humans. Nature *507*, 354–357.

7. Vernot, B., and Akey, J.M. (2014). Resurrecting surviving Neandertal lineages from modern human genomes. Science *343*, 1017–1021.

8. Sankararaman, S., Patterson, N., Li, H., Pääbo, S., and Reich, D. (2012). The Date of Interbreeding between Neandertals and Modern Humans. PLoS Genet *8*, e1002947.

9. Fu, Q., Li, H., Moorjani, P., Jay, F., Slepchenko, S.M., Bondarev, A.A., Johnson, P.L.F., Aximu-Petri, A., Prufer, K., de Filippo, C., et al. (2014). Genome sequence of a 45,000-year-old modern human from western Siberia. Nature *514*, 445–449.

10. Seguin-Orlando, A., Korneliussen, T.S., Sikora, M., Malaspinas, A.-S., Manica, A., Moltke, I., Albrechtsen, A., Ko, A., Margaryan, A., Moiseyev, V., et al. (2014). Genomic structure in Europeans dating back at least 36,200 years. Science *346*, 1113–1118.

11. Green, R.E., Malaspinas, A.S., Krause, J., Briggs, A.W., Johnson, P.L.F., Uhler, C., Meyer, M., Good, J.M., Maricic, T., Stenzel, U., et al. (2008). A Complete Neandertal Mitochondrial Genome Sequence Determined by High-Throughput Sequencing. Cell *134*, 416–426.

12. Briggs, A.W., Good, J.M., Green, R.E., Krause, J., Maricic, T., Stenzel, U., Lalueza-Fox, C., Rudan, P., Brajković, D., Kućan, Ž., et al. (2009). Targeted Retrieval and Analysis of Five Neandertal mtDNA Genomes. Science *325*, 318–321.

13. Wood, R.E., Higham, T.F.G., De Torres, T., Tisnérat-Laborde, N., Valladas, H., Ortiz, J.E., Lalueza-Fox, C., Sánchez-Moral, S., Cañaveras, J.C., Rosas, A., et al. (2013). A new date for the neanderthals from el Sidrón cave (Asturias, northern Spain). Archaeometry *55*, 148–158.

14. Castellano, S., Parra, G., Sánchez-Quinto, F. a, Racimo, F., Kuhlwilm, M., Kircher, M., Sawyer, S., Fu, Q., Heinze, A., Nickel, B., et al. (2014). Patterns of coding variation in the complete exomes of three Neandertals. Proc. Natl. Acad. Sci. U. S. A. *111*, 6666–6671.

15. Karmin, M., Saag, L., Vicente, M., Sayres, M.A.W., Järve, M., Talas, U.G., Rootsi, S., Ilumäe, A.-M., Mägi, R., Mitt, M., et al. (2015). A recent bottleneck of Y chromosome diversity coincides with a global change in culture. Genome Res. *25*, 459–466.

16. Mendez, F.L., Krahn, T., Schrack, B., Krahn, A.M., Veeramah, K.R., Woerner, A.E., Fomine, F.L.M., Bradman, N., Thomas, M.G., Karafet, T.M., et al. (2013). An African American paternal lineage adds an extremely ancient root to the human y chromosome phylogenetic tree. Am. J. Hum. Genet. *92*, 454–459.

17. Li, H. (2011). A statistical framework for SNP calling, mutation discovery, association mapping and population genetical parameter estimation from sequencing data. Bioinformatics *27*, 2987–2993.

18. Kent, W.J. (2013). http://hgdownload.soe.ucsc.edu/goldenPath/hg19/vsPanTro4/axtNet.

19. Poznik, G.D., Henn, B.M., Yee, M.-C., Sliwerska, E., Euskirchen, G.M., Lin, A.A., Snyder, M., Quintana-Murci, L., Kidd, J.M., Underhill, P.A., et al. (2013). Sequencing Y Chromosomes Resolves Discrepancy in Time to Common Ancestor of Males Versus Females. Science *341*, 562–565.

20. Skaletsky, H., Kuroda-Kawaguchi, T., Minx, P.J., Cordum, H.S., Hillier, L., Brown, L.G., Repping, S., Pyntikova, T., Ali, J., Bieri, T., et al. (2003). The male-specific region of the human Y chromosome is a mosaic of discrete sequence classes. Nature *423*, 825–837.

21. Rasmussen, M., Anzick, S.L., Waters, M.R., Skoglund, P., DeGiorgio, M., Stafford Jr, T.W., Rasmussen, S., Moltke, I., Albrechtsen, A., Doyle, S.M., et al. (2014). The genome of a Late Pleistocene human from a Clovis burial site in western Montana. Nature *506*, 225–229.

22. Adzhubei, I., Jordan, D.M., and Sunyaev, S.R. (2013). Predicting Functional Effect of Human Missense Mutations Using PolyPhen-2. Curr. Protoc. Hum. Genet. *76*, 1–41.

23. Schwarz, J.M., Cooper, D.N., Schuelke, M., and Seelow, D. (2014). MutationTaster2: mutation prediction for the deep-sequencing age. Nat Meth *11*, 361–362.

24. Nordborg, M. (1998). On the probability of Neanderthal ancestry. Am. J. Hum. Genet. *63*, 1237–1240.

25. Williams, N.A., Close, J.P., Giouzeli, M., and Crow, T.J. (2006). Accelerated evolution of Protocadherin11X/Y: A candidate gene-pair for cerebral asymmetry and language. Am. J. Med. Genet. Part B Neuropsychiatr. Genet. *141B*, 623–633.

26. Lee, K.H., Song, G.J., Kang, I.S., Kim, S.W., Paick, J.S., Chung, C.H., and Rhee, K. (2003). Ubiquitin-specific protease activity of USP9Y, a male infertility gene on the Y chromosome. Reprod. Fertil. Dev. *15*, 129–133.

27. Tyler-Smith, C., and Krausz, C. (2009). The Will-o'-the-Wisp of Genetics—Hunting for the Azoospermia Factor Gene. N. Engl. J. Med. *360*, 925–927.

28. Wong, H., Wang, G., Croessmann, S., Zabransky, D., Chu, D., Garay, J., Cidado, J., Cochran, R., Beaver, J., Aggarwal, A., et al. (2015). TMSB4Y is a candidate tumor suppressor on the Y chromosome and is deleted in male breast cancer. Oncotarget *6*, 44927–44940.

29. Li, N., Dhar, S.S., Chen, T.-Y., Kan, P.-Y., Wei, Y., Kim, J.-H., Chan, C.-H., Lin, H.-K., Hung, M.-C., and Lee, M.G. (2016). JARID1D is a suppressor and prognostic marker of prostate cancer invasion and metastasis. Cancer Res. *76*, 831–843.

30. Nielsen, H.S. (2011). Secondary recurrent miscarriage and H-Y immunity. Hum. Reprod. Update *17*, 558–574.

31. Bogaert, A.F., and Skorska, M. (2011). Frontiers in Neuroendocrinology Sexual orientation, fraternal birth order, and the maternal immune hypothesis: A review. Front. Neuroendocrinol. *32*, 247–254.

32. Wang, W., Meadows, L.R., den Haan, J.M., Sherman, N.E., Chen, Y., Blokland, E., Shabanowitz, J., Agulnik, A.I., Hendrickson, R.C., Bishop, C.E., et al. (1995). Human H-Y: a male-specific histocompatibility antigen derived from the SMCY protein. Science *269*, 1588–1590.

33. Haldane, J.B.S. (1922). Sex ratio and unisexual sterility in hybrid animals. J. Genet. *12*, 101–109.

Figura 1. Árbol de inferencia. **a**, A priori, cualquiera de tres árboles podrían haber relacionado los cromosomas Y de chimpancé (Chimp), neandertal (Neander), A00 haplogrupo, y la referencia humana (Ref). Las mutaciones en la rama a apoyan la topología *i*, con el linaje neandertal como grupo externo al de los humanos modernos, mientras que las mutaciones en las ramas b y c apoyan las topologías *ii* y *iii*, respectivamente. Ramas d, e, y f corresponden a mutaciones particulares a los distintos linajes. **b**, Recuento de SNVs consistentes con cada rama. Las columnas se refieren a los conjuntos de coordenadas considerados (**Materiales y Métodos)**. Sitios incompatibles son aquellos que no se pueden explicar por una sola mutación en cualquiera de los tres árboles.

Figura 2. Estimación de la TACMR del cromosoma Y de neandertal y moderno. La cantidad de interés principal es $T_{NR} = T_{NM} + T_{AR}$. Las ramas están etiquetadas como en la **Figura 1**, y 'M' indica el ancestro común más reciente de los linajes humanos modernos.

Figura 3. Relación del cromosoma Y de neandertal y los de los humanos modernos. La genealogía (árbol rojo) se puede explicar parsimoniosamente como reflejo de la divergencia de la poblaciones (árbol gris). No encontramos ninguna evidencia de: (a) un origen súper arcaico muy divergente del cromosoma Y de neandertal, (b) antiguo flujo génico con posterioridad a la división de las poblaciones, o (c) introgresión relativamente reciente de un cromosoma Y humano moderno en la población neandertal.

Tabla 1. Mutaciones que cambian las secuencias proteicas[1].

Coordenada	Gen	Linaje[2]	Sustitución[3]	Efecto[4]	Herramienta[5]	Función	MIM[6]
2,844,774	*ZFY*	N	p.Val140Ala	B	P2	potencial factor de transcripción	490000
			p.Val331Ala	B	P2		
2,847,322	*ZFY*	A	p.Ile374Thr	B	P2	"	
			p.Ile488Thr	**PrD**	P2		"
			p.Ile565Thr	B	P2		
4,967,724	*PCDH11Y*[7]	N	p.Lys702Thr	B, B	P2, MT	protocadherina	400022
5,605,569	*PCDH11Y*[7]	N	p.Ser1203Arg	**PrD**	P2	“	“
6,932,032	*TBL1Y*	N	p.Gly100Ala	B, B	P2,MT		400033
14,832,610	*USP9Y*	N	p.Glu62Gly	**PrD**	P2	peptidasa	400005
14,832,620	*USP9Y*	R	p.Glu65Asp	B	P2	"	"
14,838,553	*USP9Y*	N	p.Ala162Thr	B	P2	"	"
15,816,262	*TMSB4Y*	N	p.Ser16*	**PrD**	MT	secuestro de actina	400017
21,868,167	*KDM5D*	R, A	p.Arg1445Gln	B, B	P2, MT	demetilasa	
			p.Arg1388Gln	B, B	P2, MT		426000
			p.Arg1476Gln	B, B	P2, MT		
21,905,071	*KDM5D*	R, A	p.Ile69Val	**PoD**, B	P2, MT	"	"
23,545,399	*PRORY*	A	p.Arg125Cys				

[1] Por favor ver **Tabla S3** y los Datos Suplementarios para información adicional sobre todas las mutaciones.
[2] Linaje(s) que tienen los alelos derivados. N, neandertal; A, A00; R, referencia.
[3] Múltiples listados de una sola coordenada reflejan sustituciones en diferentes transcriptos del gen.
[4] B, Benigno; PoD, Posiblemente dañino; PrD, Probablemente dañino.
[5] P2, PolyPhen-2 (ancestrales a derivados); MT, MutationTaster (referencia a alternativo).
[6] Herencia mendeliana en el Hombre (Mendelian Inheritance in Man).
[7] Por favor ver **Apéndice B**.

a

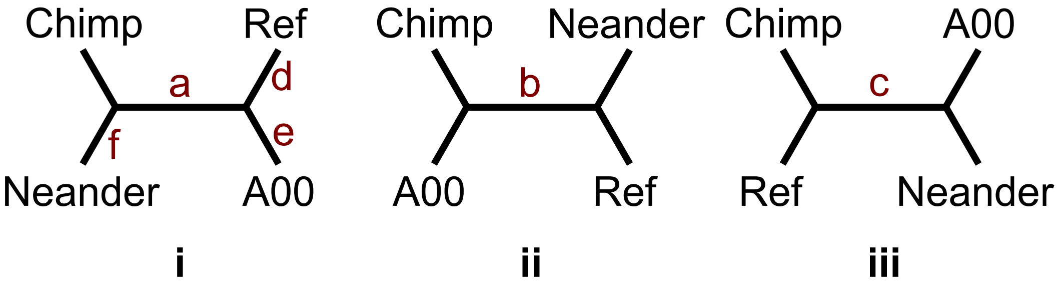

b

	Filtro 1	Filtro 2	Filtro 3	Filtro 4
**Sitios examinables**	118,643	100,324	80,420	75,596
**Sitios variables**	146	126	81	74
**Rama**				
a	24	20	13	12
b	4	4	4	4
c	0	0	0	0
d	16	14	13	11
e	22	20	17	16
f	77	66	33	30
Incompatibles	3	2	1	1

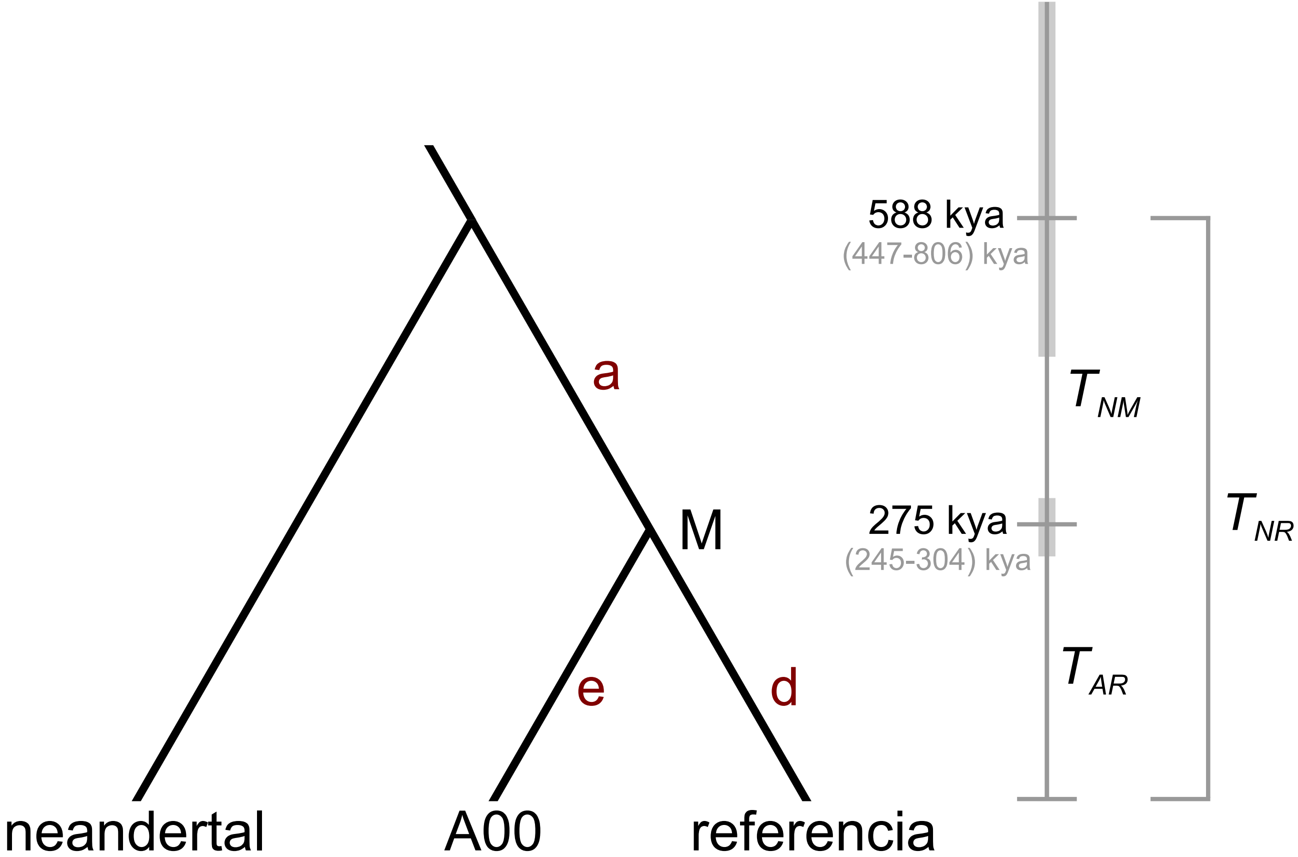

a
M
e
d
neandertal
A00
referencia
588 kya
(447-806) kya
275 kya
(245-304) kya
T_{NM}
T_{AR}
T_{NR}

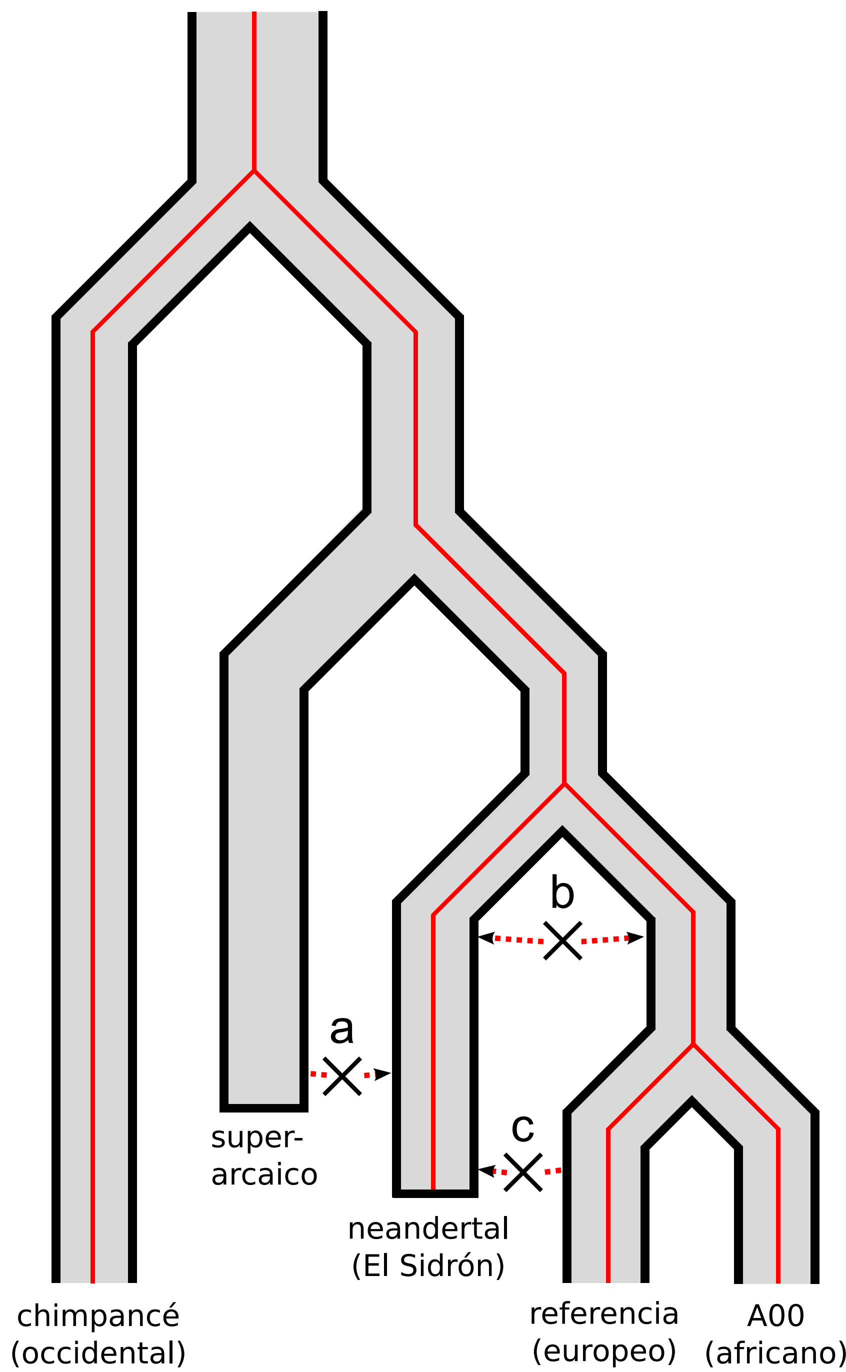

b
a
c
super-
arcaico
neandertal
(El Sidrón)
chimpancé
(occidental)
referencia
(europeo)
A00
(africano)

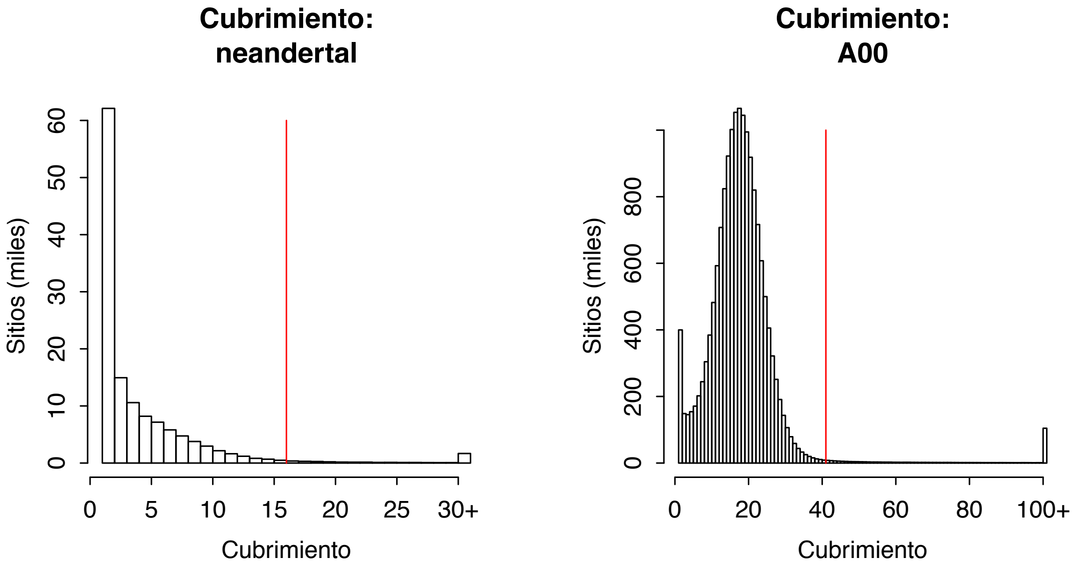

Figura S1. Cubrimiento de secuencia. Las líneas rojas indican valores 5 desviaciones estándar por encima de las medias. Descartamos aquellos sitios con cubrimientos que excedían estos valores.

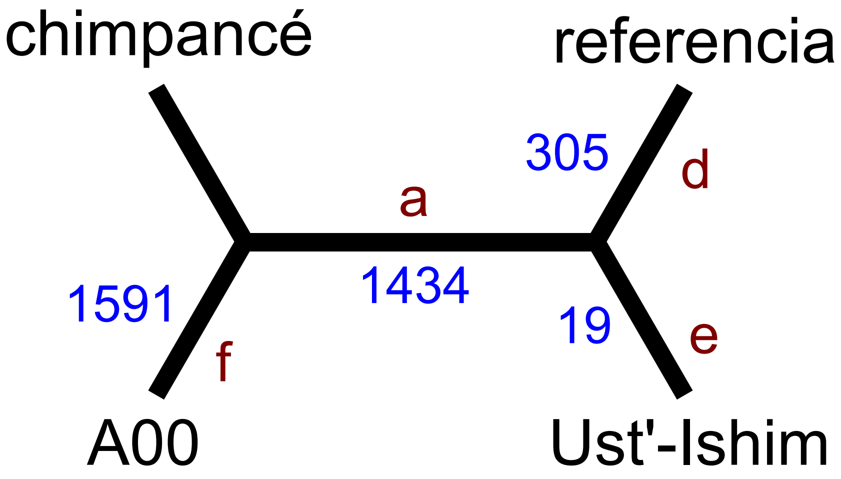

Figura S2. Estimando T_{AR}. Los números en azul indican la cantidad de mutaciones en los ~7.83 Mb de secuencia donde se superponen.
